# B Lymphocytes Are a Predictive Marker of Eribulin Response and Overall Survival in Locally Advanced or Metastatic Breast Cancer: A Multicenter, Two-Cohort, Non-Randomized, Open-Label, Retrospective Study

**DOI:** 10.3389/fonc.2022.909505

**Published:** 2022-06-23

**Authors:** Liubov A. Tashireva, Nataliya O. Popova, Anna Yu. Kalinchuk, Viktor E. Goldberg, Elena I. Kovalenko, Elena V. Artamonova, Aleksey G. Manikhas, Dmitriy M. Ponomarenko, Nataliya V. Levchenko, Elena I. Rossokha, Svetlana Yu. Krasilnikova, Marina A. Zafirova, Evgeniy L. Choynzonov, Vladimir M. Perelmuter

**Affiliations:** ^1^ Research Institute of Oncology, Tomsk National Research Medical Center, Russian Academy of Sciences, Tomsk, Russia; ^2^ Federal State Budgetary Institution National Medical Research Center of Oncology of the Ministry of Health of the Russian Federation (NN Blokhin NMRCO), Moscow, Russia; ^3^ Saint-Petersburg State Budgetary Healthcare Institution “City Clinical Oncology Center” (1st Oncology (Surgery) Department, Saint-Petersburg, Russia; ^4^ Chemotherapy Department, Irkutsk Regional Cancer Center, Irkutsk, Russia; ^5^ Department of Oncology, Irkutsk State Medical Academy of Postgraduate Education, Irkutsk, Russia; ^6^ Chemotherapy Department, State Budgetary Healthcare Institution “St. Petersburg Clinical Scientific and Practical Center of Specialized Types of Medical Care (Oncological)”, St. Petersburg, Russia; ^7^ Chemotherapy Department, Regional State Budgetary Healthcare Institution “Altai Regional Oncology Center”, Barnaul, Russia; ^8^ Chemotherapy Department, Sverdlovsk Regional Oncological Dispensary, Ekaterinburg, Russia; ^9^ Federal State Budget Educational Institution of Higher Education, Ural State Medical University of the Ministry of Health of the Russian Federation, Ekaterinburg, Russia

**Keywords:** triple negative breast cancer, eribulin response, tumor microenvironment, B cell, overall survival

## Abstract

Triple-negative breast cancer has no specific treatment and unfavorable prognosis. Eribulin is one of the drugs widely used in this cohort of patients. In addition to its antimitotic effect, eribulin has an immunomodulant effect on the tumor microenvironment. In this study, we discover immunological markers, such as tumor-infiltrating lymphocytes, CD8+, CD4+, FoxP3+, CD20+ lymphocytes, and their PD1 positivity or negativity, with the ability to predict benefits from eribulin within locally advanced or metastatic triple-negative breast cancer. The primary objective was to explore the association of composition of immune cells in the microenvironment with response to eribulin. The key secondary objective was overall survival. Seven-color multiplex immunofluorescence was used to phenotype lymphocytes in the primary tumor. It has been shown that the PD1-negative-to-PD1-positive B cells ratio in primary tumors more than 3 is an independent predictor of the short-term effectiveness of eribulin [OR (95%CI) 14.09 (1.29-153.35), p=0.0029] and worse overall survival [HR (95%CI) 11.25 (1.37-70.25), p=0.0009] in patients with locally advanced or metastatic triple-negative breast cancer.

## Introduction

The main cytotoxic mechanism of action of eribulin mesylate (eribulin) is the stoppage of cell division by inhibiting the elongation of microtubules (1). It should be noted that the mechanism of action of eribulin differs from that of other antimitotic agents, such as taxanes or derivatives of vinca alkaloids (2). Eribulin binds to the ends of microtubules and inhibits their polymerization. Taxanes, on the other hand, act inside microtubules and cause their superstabilization (i.e., forced elongation). The advantage of this mechanism of action still remains unclear (3). In addition to the antimitotic mechanism of action of eribulin, there are other mechanisms of action. Thus, experimental models and cell cultures have shown eribulin-mediated suppression of the epithelial-mesenchymal transition (EMT) of tumor cells (4), as well as stimulation of vascular tumor remodeling (5). The effects of eribulin on the tumor microenvironment have also been described. The most common indicator for monitoring the immune response, i.e., the percentage of tumor-infiltrating lymphocytes (TIL) in the tumor stroma, has been described as a prognostic factor and predictor of eribulin effectiveness. Thus, patients with triple-negative breast cancer who have a high TIL level demonstrated a significantly longer relapse-free survival than patients with low TILs, while no significant differences in the duration of relapse-free survival regardless of the molecular genetic type of BC or in patients with BC other than triple-negative type were found (6). The effect of eribulin on PD-L1 status and expression of lymphocytic markers was demonstrated. Eribulin response was significantly associated with a change in the pattern of expression of PD-L1 and FoxP3 to the opposite (7). Evidence that a possible mechanism of the antitumor action of eribulin is the modification of the tumor microenvironment allows us to consider the parameters of the microenvironment as possible predictors of eribulin response. The aim of the study was to identify immunological predictors of eribulin response and overall survival among locally advanced or metastatic triple-negative breast cancer patients.

## Methods

### Study Design and Participants

**Figure 1:**
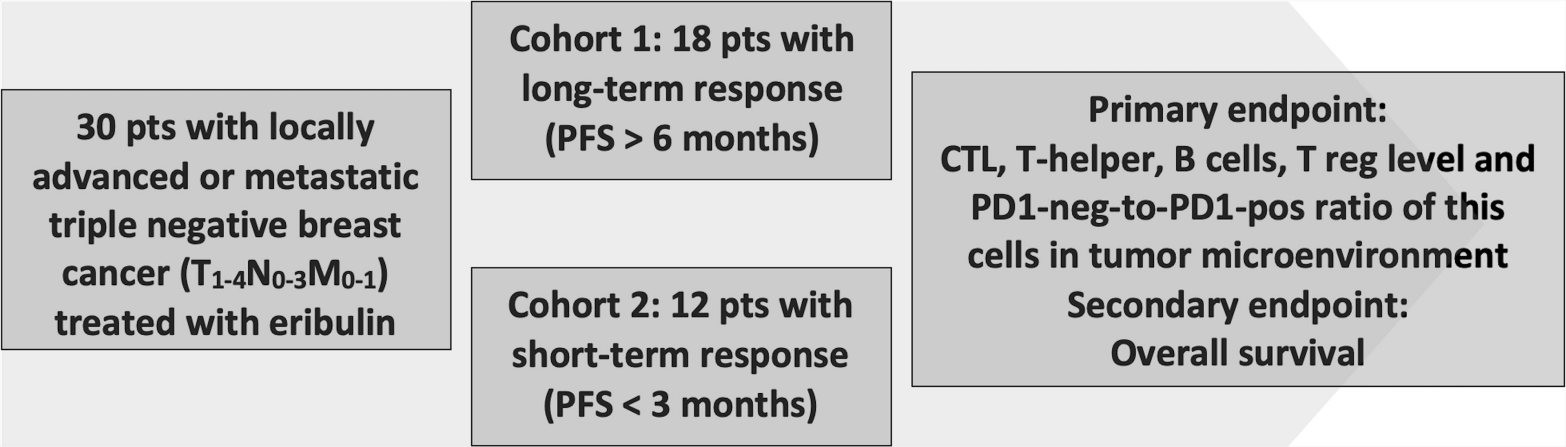
Study design.

In an open retrospective, multicenter, observational, non-randomized study 30 triple-negative breast cancer (TNBC) patients were enrolled and treated with eribulin at the Cancer Research Institute (Tomsk National Research Medical Center), St. Petersburg Clinical Scientific and Practical Center of Specialized Types of Medical Care (Oncological), Sverdlovsk Regional Oncological Dispensary, Altai Regional Oncology Center, Irkutsk Regional Oncological Dispensary, State Budgetary Healthcare Institution “City Clinical Oncology Center” (St. Petersburg). All patients gave written consent (clause 3, article 13 of the Federal Law of the Russian Federation No. 323-FZ dated November 21, 2011). The study was approved by the local ethics committee of the Tomsk NRMC Institutional Review Board (No. 7 dated April 01, 2019). Inclusion criteria: age of patients over 18 years; histological verification of the non-specific invasive breast carcinoma, triple-negative subtype, prior therapy by anthracyclines and taxane drugs in an adjuvant or metastatic treatment regimen. Cohort 1 included patients with long-term response to eribulin therapy (progression-free survival (PFS) >6 months, median 9 months) (n=18) and cohort 2 included patients with a short-term response to eribulin therapy (PFS <3 months, median 2 months) (n=12) (**Figure 1**). All patients received eribulin for advanced disease (1.4 mg/m2, 1 and 8 d of every 21-day cycle) until adverse events or progression or until death. Age, histological grade, primary tumor size, stage, and time to progression or death were obtained from the clinic–pathologic and outcome data.

### Histological Evaluation

Quantification of tumor-infiltrating lymphocytes (TILs) in tumor samples was performed using the recommendations published by TILs International Working Group 2018 (8). The cut-off value of TILs was 50% to define low and high TILs.

### Immunofluorescence Multiplex Assay Using Tyramide Signal Amplification

Analysis of the subpopulation composition of TILs and their expression of PD1 and PD-L1 proteins was performed by Immunofluorescence Multiplex Assay using Tyramide Signal Amplification. Immunostainer Bond RXm (Leica, Germany) was used to perform the multiplex staining protocol. Design and validation of protocol were performed according to recommendation (9). The following panel of antibodies were used: anti-human CD3 (Dako, Polyclonal), anti-human CD8 (Dako, C8/144B), anti-human CD20 (Dako, L26), anti-human PD1 (Abcam, NAT105), anti-human FOXP3 (Cell Marque, EP340), anti-human PD-L1 (Ventana, SP142). Tissue sections were counterstained with DAPI and mounted (Prolong antifade, Thermo Fisher Scientific, Waltham, MA, USA). The automated quantitative imaging system Vectra^®^ 3.0 (Akoya Biosciences, Marlborough, Massachusetts, USA) was used to acquire images. Whole slides were scanned at × 4 magnification and multispectral images of regions of interest (ROIs) were obtained at × 10 magnification. The number of ROIs per slide was 10. InForm^®^ software (Akoya Biosciences, USA) was used to perform image analysis. In each sample, images of the entire specimen were analyzed, except for areas with artificial staining or poor quality. The following cells were identified in the microenvironment: cytotoxic lymphocytes (CTL) – CD3+CD8+, T-helper lymphocytes (Th cell) – CD3+CD8-, T regulatory lymphocytes (Treg) – CD3+FoxP3+ and B lymphocytes – CD3-CD20+. PD1 expression was evaluated in each cell type. The number of cells was calculated as % of all TILs. PD-L1 expression in ICs was scored in the IHC simulation regimen according to published guidelines (10). Evaluation of TILs by MIF was performed as described (11).

### Statistical Analysis

To compare the differences in independent nonparametric variables between two groups we used the Mann-Whitney test. For categorical variables, Fisher’s exact test was used. ROC analysis was used to assess the predictive effectiveness of the marker (the area under the curve (AUC) and 95% confidence interval (95%CI), as well as the values of sensitivity and specificity, were calculated). The predictive significance of the parameters was assessed using univariate and multivariate regression analysis (Odds ratios (ORs) and 95%CI were calculated). Univariate and multivariate Cox-regression was used to compute Hazard Ratio (HR) and 95%CI for each variable. Overall survival (OS) was defined as the date of death and patients still alive were censored at the date of the last visit. Kaplan-Meier method was used to establish survival curves. The Kaplan–Meier estimates are provided together with bootstrap confidence intervals (1000 samples). All p-values were two-sided, and values less than 0.05 were considered significant. Statistical analysis was performed using Prism 9 (GraphPad, USA) and Statistica 12.0 (StatSoft, USA) software.

## Results

### Patient Characteristics

Thirty TNBC patients were enrolled. Clinic-pathological characteristics by each cohort are given in **Table 1**. Patients with short-term and long-term responses to eribulin were comparable by clinical characteristics and pathological tumor parameters (**Table 1**).

**Table 1:** Patient characteristics (N=30, TNBC).

Parameter		Long-term response	Short-term response
		(N=18)	(N=12)
Age		59 (37–80)	55 (27–76) p=0.126
Primary tumor size (mm)		25 (14-50)	31 (15-49) p=0.253
Ki67 level	<50	8 (44%)	7 (58%)
	>50	10 (56%)	5 (42%) p=0.710
Lymph node metastasis	no	10 (56%)	6 (50%)
	yes	8 (44%)	6 (50%) p>0.999
Neoadjuvant chemotherapy	no	8 (44%)	4 (33%)
	yes	10 (56%)	8 (67%) p=0.708
Menopausal status	preserved	8 (44%)	6 (50%)
	menopause	10 (56%)	6 (50%) p>0.999
Grade	1	0 (0%)	0 (0%)
	2	5 (28%)	2 (17%)
	3	13 (72%)	10 (83%) p=0.669
Stage	I	0 (0%)	0 (0%)
	II	8 (45%)	3 (25%) p=0.442
	III	6 (33%)	3 (25%) p=0.703
	IV	4 (22%)	6 (50%) p=0.139
Distant metastases		9 (50%)	5 (42%) p=0.722

### Evaluation of TILs and PD-L1 Status

No significant difference was found in TIL levels depending on eribulin response. TIL levels in the long-term response cohort was 27.95 (13.60-45.08) and in short-term response cohort 23.87 (5.09-43.95) (p=0.4584). Also, eribulin response had no association with PD-L1 status (cut-off 1%). In the long-term response cohort 44.4% (8/18) of cases were PD-L1 positive and the in short-term response cohort – 50.0% (6/12) (p>0.9999).

### Immune Cells Phenotyping

**Figure 2:**
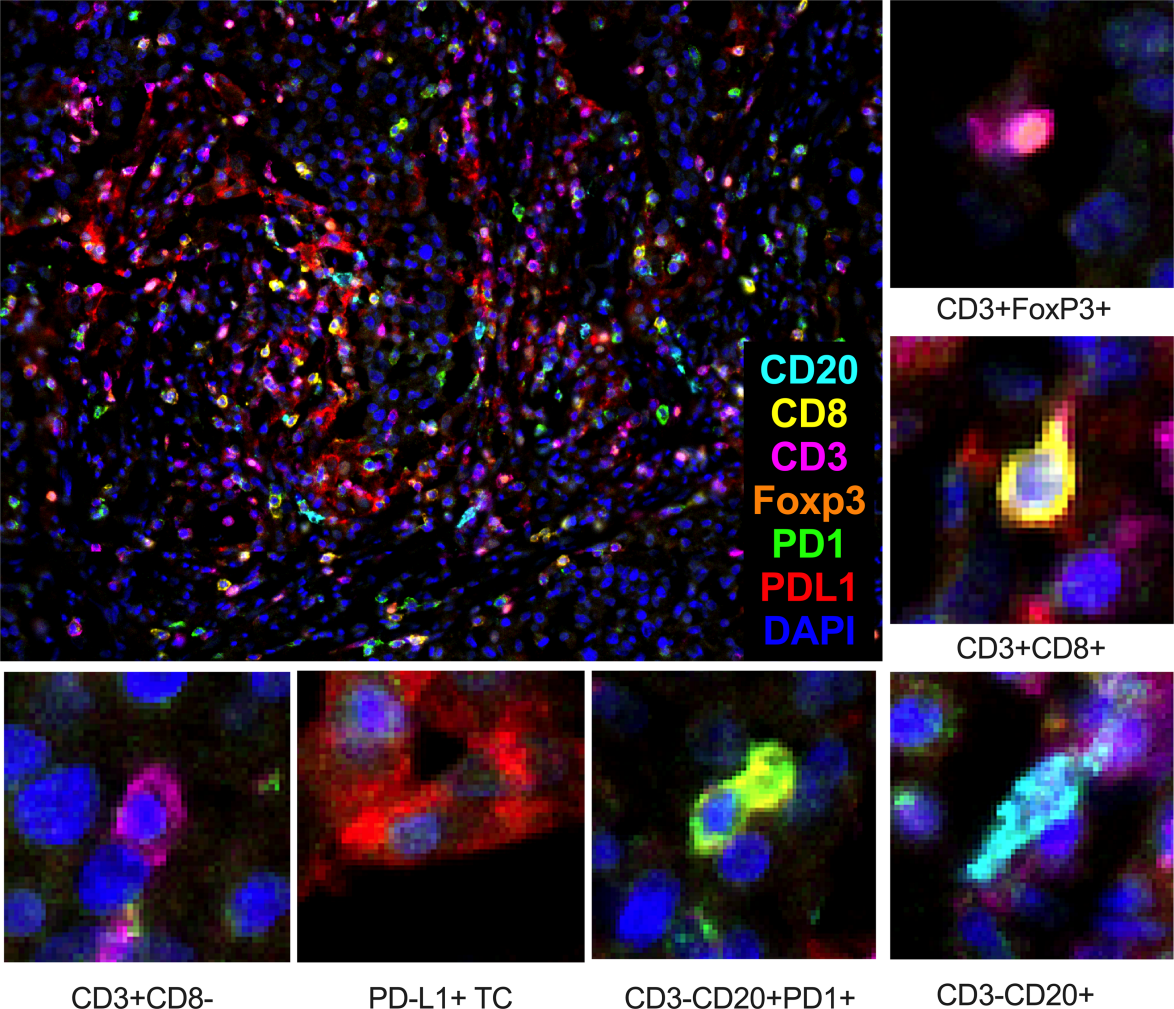
Composition of cytotoxic lymphocytes (CTL – CD3+CD8+), T regulatory (Treg – CD3+FoxP3+) lymphocytes, T-helper (Th – CD3+CD8-) lymphocytes, and B lymphocytes (CD3-CD20+) in the tumor microenvironment of TNBC patients. TC – tumor cell. Seven-color immunofluorescence, magnification 200x.

We identify four subpopulations of immune cells: cytotoxic lymphocytes (CTL), T regulatory (Treg) lymphocytes, T helper (Th) lymphocytes, and B lymphocytes (**Figure 2**). Each cell was characterized by PD1 expression. No cell subpopulation frequencies or PD1 expression in examined lineage were found associated with eribulin response in TNBC. CTL, T cell, and Treg were found in all cases. B lymphocytes were found in cohort 1 in 88.8% (16/18) of cases and in cohort 2 in 100% (12/12) of cases, p=1.000).

The number of examined subsets of lymphocytes did not differ depending on eribulin response (**Table 2**).

**Table 2:** The number of immune cell subsets depending on eribulin response, Me (Q1-Q3).

Lymphocyte subtype	PD1 expression	Eribulin response		p-value	
		Long-term	Short-term		
Cytotoxic	Neg	6.01 (0.85-11.27)	5.85 (0.16-42.32)	0.6615	
	Pos	1.16 (0.30-5.11)	2.32 (0.04-6.77)	0.8840	
T regulatory	Neg	0.96 (0.32-2.93)	2.72 (0.86-6.08)	0.0849	
	Pos	0.74 (0.13-3.19)	1.33 (0.43-2.49)	0.5102	
T-helper	Neg	8.30 (0.89-16.23)	4.83 (2.40-12.36)	0.8593	
	Pos	2.40 (0.30-9.28)	2.17 (1.09-4.60)	0.9419	
B	Neg	1.42 (0.32-3.19)	2.32 (0.47-4.62)	0.3911	
	Pos	0.81 (0.11-3.01)	0.35(0.01-1.93)	0.3511	

**Figure 3:**
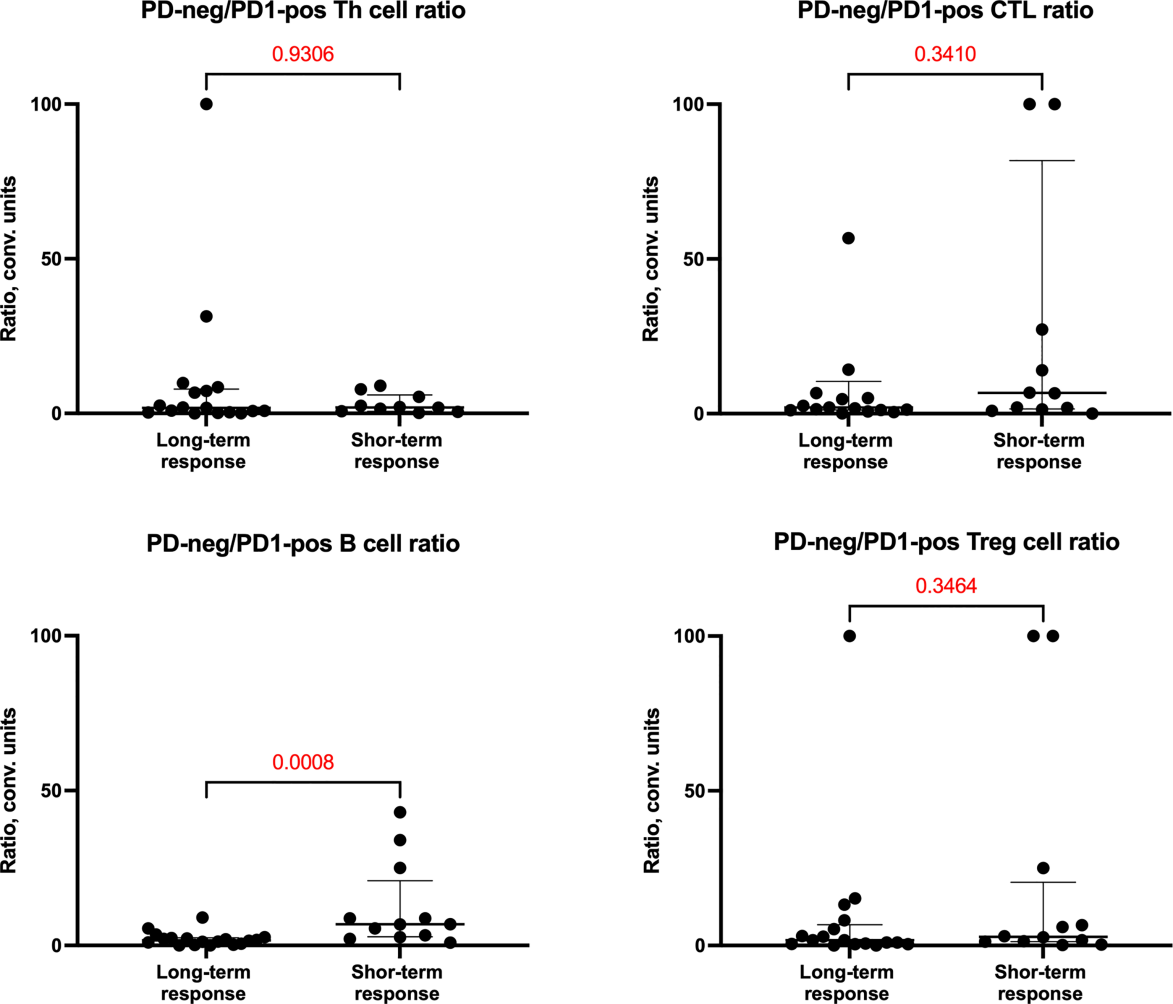
PD1-neg/PD1-pos cell ratio of cells in tumor microenvironment depending on the response of patients to eribulin therapy.

We also evaluated cellular ratios to evaluate for eribulin response-related shifts (**Figure 3**) and identified a reduction in PD1-neg/PD1-pos B cell ratios in the long-term response cohort.

**Figure 4:**
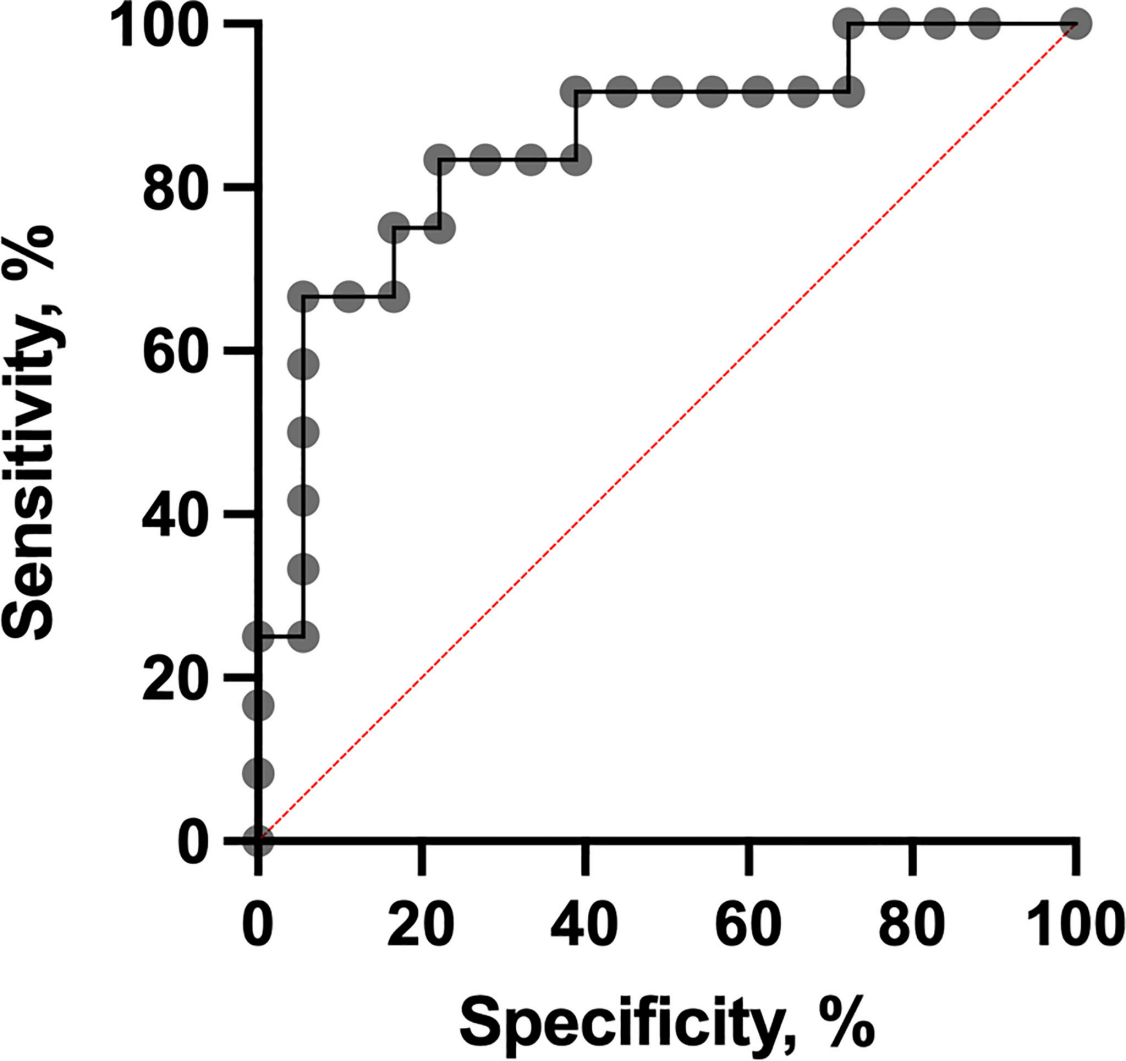
The ROC curve of the PD1-neg/PD1-pos B cell ratio for predicting eribulin response.

It was shown that PD1-neg/PD1-pos B cell ratios were higher in the short-term eribulin response cohort versus the long-term eribulin response cohort (6.82 (2.83-20.94) and 1.65 (0.49-2.49), respectively, p=0.0008). To assess the predictive value of PD1-neg/PD1-pos B cell ratio and cut-off, ROC analysis was performed (**Figure 4**).

**Figure 5:**
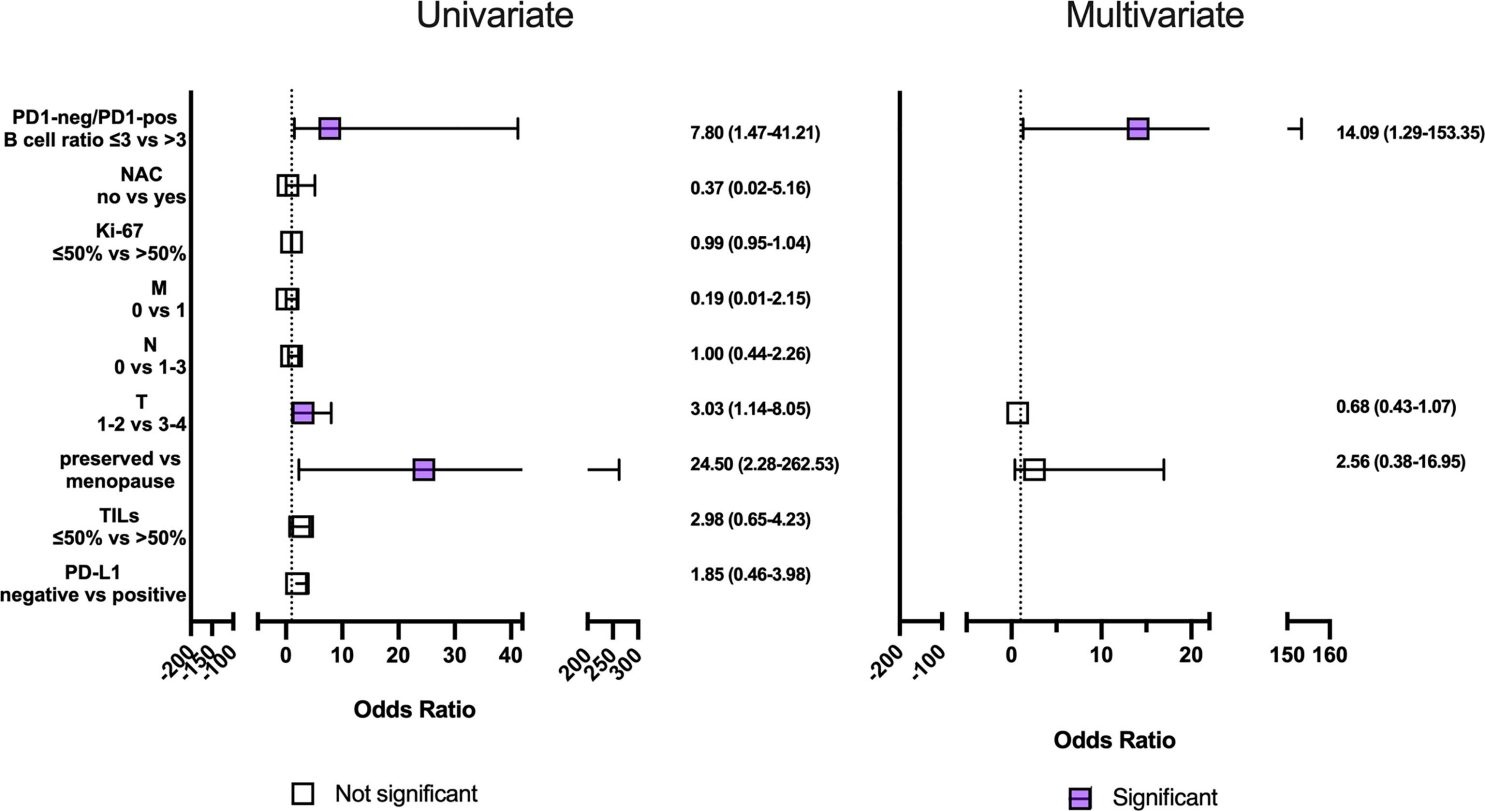
Univariate and multivariate regression analysis clinic, pathologic and immunologic parameters to short-term eribulin response prediction in locally advanced or metastatic triple-negative breast cancer patients. Horizontal bars represent the 95% confidence intervals (CI) of Odds ratios. Statistically significant variables are shown in purple.

The PD1-neg/PD1-pos B cell ratio cut-off value was 3 (area under the ROC curve [AUC], 0.85 (95% CI 0.70-0.99; p=0.0013) with 75.0% sensitivity and 83.3% specificity. In patients who had PD1-neg/PD1-pos B cell ratio in the primary tumor tissue more than 3 in 75.0% (9/12) of cases, short-term eribulin response was observed, while in patients less than 3 in 16.6% (3/18) of cases the short-term effect was registered (p=0.0024). So, the relative risk of a short-term eribulin response, if PD1-neg/PD1-pos B cell ratio is more than 3, was 3.33 (95% CI 1.47-9.51). Univariate and multivariate logistic regressionanalysise was used to assess the predictive value of clinic, pathologic, and immunologic parameters (**Figure 5**). The parameters studied have been consistently tested as predictors of eribulin therapy effectiveness in univariate regression analysis. Subsequently, all parameters that showed their statistical significance were included in multivariate analysis to confirm predictive consistency.

Univariate regression analyses revealed an effect on the short-term eribulin response prediction of the menopause, T3-4, and PD1-neg/PD1-pos B cell ratio of more than 3. However, in multivariate analysis only PD1-neg/PD1-pos B cell ratio of more than 3 was an independent predictive factor (p=0.0029).

### Overall Survival Analysis

Univariate Cox-regression analysis revealed an association of T3-4 (p=0.0400) and PD1-neg/PD1-pos B cell ratio >3 (p=0.0028) with worse overall survival (**Table 3**). But in multivariate analysis only PD1-neg/PD1-pos B cell ratio >3 (p=0.0009) was an independent prognostic factor for worse overall survival in TNBC patients.

**Table 3:** Univariate and multivariate Cox-regression analysis of overall survival in patients with locally advanced or metastatic triple-negative breast cancer.

Parameter		Univariate analysis			Multivariate analysis			
		OR	95% CI	P		OR	95% CI	p
Menstrual function	preserved	1						
	menopause	1.66	0.43-6.28	0.4553			–	
T	1-2	1						
	3-4	4.15	1.06-16.21	0.0400		3.08	0.76-19.12	0.1013
N	0	1						
	1-3	1.67	0.47-5.97	0.4263		–		
M	0	1						
	1	1.22	0.26-5.79	0.7943		–		
Ki67	<50	1						
	>50	0.79	0.95-2.35	0.2647		–		
NAC	no	1						
	yes	0.59	0.07-3.92	0.3378		–		
TILs	Low	1						
	High	0.17	0.12-11.93	0.6667		–		
PD-L1	Negative	1						
	Positive	1.69	0.79-4.28	0.3637		–		
PD1-neg/PD1-pos B cell ratio	≤3	1						
	>3	7.60	1.60-36.00	0.0028		11.25	1.37-70.25	0.0009

**Figure 6:**
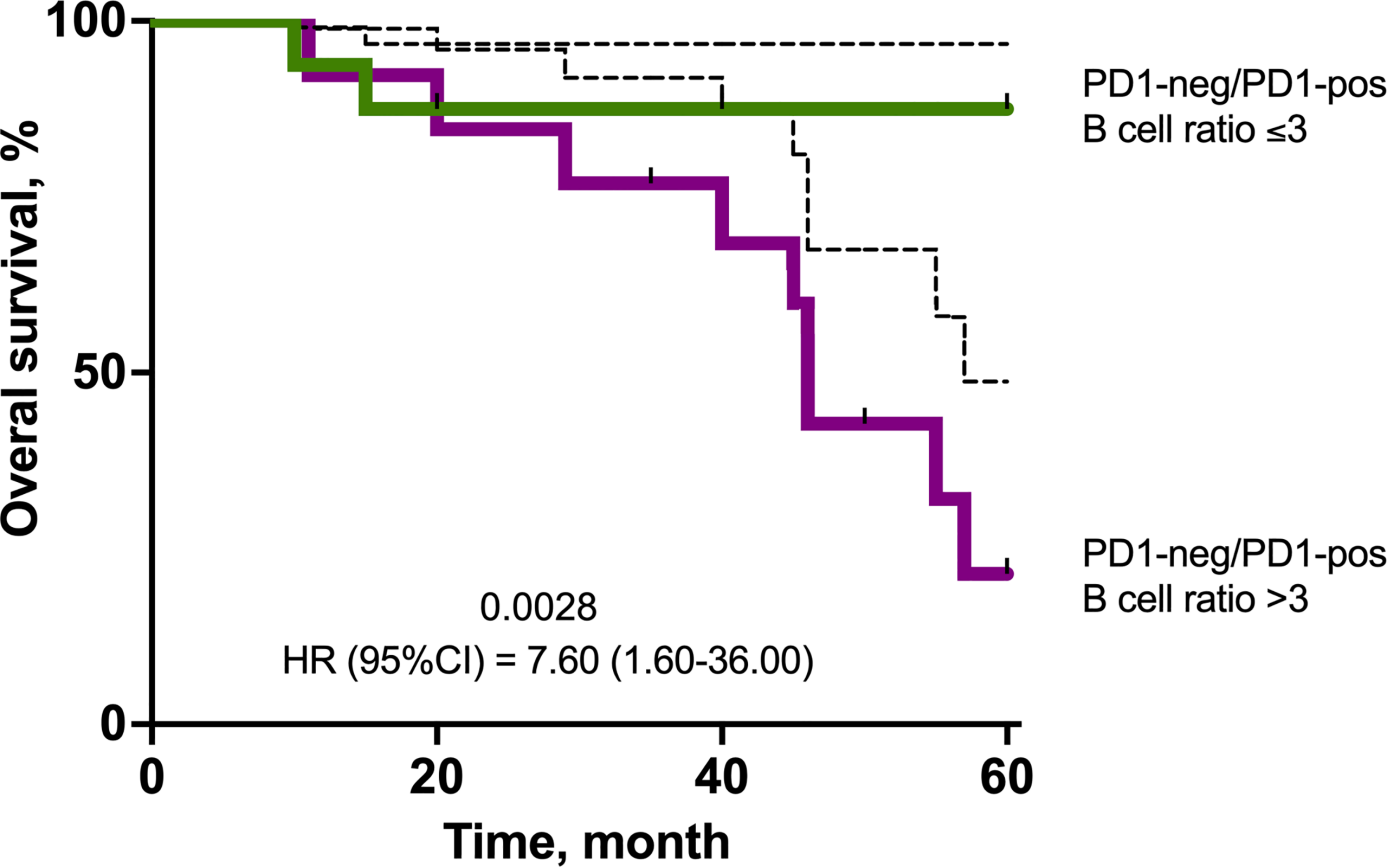
Kaplan–Meier survival plot showing the overall survival of TNBC patients in the cohort stratified by eribulin response. The estimated overall survival values are provided together with bootstrap confidence intervals (dotted line).

Patients who had PD1-neg/PD1-pos B lymphocyte ratio >3 in primary tumor had significantly worse OS. Survival rate in PD1-neg/PD1-pos B lymphocyte ratio >3 was 21% versus 87% in PD1-neg/PD1-pos B lymphocyte ratio ≤3 (p=0.0028) (**Figure 6**).

## Discussion

The search for additional criteria for predicting the effectiveness of eribulin therapy in patients with breast cancer is urgent. In our study, we evaluated the predictive significance of immunological parameters of the tumor microenvironment as markers of the therapeutic effectiveness of eribulin in patients with locally advanced or metastatic triple-negative breast cancer. It is known that a high level of TILs in patients with triple-negative breast cancer is associated with better overall survival and disease-free survival (12). Moreover, a similar relationship is valid for TILs expressing PD-1. It is believed that the expression of PD1 indicates an increased immunological antitumor activity and is a favorable prognostic marker (13). However, in our study, the level of TILs did not differ in patients with a long-term and short-term response to eribulin therapy, just as the frequency of occurrence of various subpopulations of lymphocytes did not differ, the ratio of PD1-negative to PD1-positive B lymphocytes was important. PD1-negative-to-PD1-positive B cells ratio of more than 3 in the primary tumor was an independent predictor of a short-term response to eribulin therapy in patients with locally advanced or metastatic triple-negative breast cancer. Results show that patients with short-term eribulin response have more negative B lymphocytes in the primary tumor. At the same time, the total number of B lymphocytes was comparable. This characteristic preceded treatment with eribulin, which means that a low level of PD1 negative B lymphocytes is a necessary condition for the implementation of the mechanisms of immunomodulation with eribulin. It has been shown that PD1 expression accompanies the activation of B cells. However, it is known that PD-1 is a negative regulator of B-cell proliferation, differentiation, and isotype class switch (14). In addition, it has been shown that PD-1/PD-L1 blockade restores IL-6 production and B-cells proliferation (15), i.e., PD-1 negative B cells are immature, more proliferating, not susceptible to PD-L1/PD1 blockade, and may ultimately lead to successful type 2 immune response.

Type 2 immune response-associated cytokines have also been shown to stimulate chronic inflammation and diminished antitumor responses (16). Current data suggest that tumor-infiltrating B cells are a favorable prognostic biomarker in breast cancer (17). In our study, the ratio of PD-1 negative to PD-1 positive B lymphocytes was decisive. Our study shows that when there are many such B cells, eribulin is more likely to be ineffective and the overall survival in a cohort of such patients will be worse. Considering that one of the proposed mechanisms of eribulin is the conversion of PD-L1 expression, that is, the effect is very similar to the effect of PD-1/PD-L1 inhibitors, then against the background of a high level of PD-1 negative B cells, eribulin will not be effective, since B cells are unable to perceive the PD-L1 signal. The identification of such cells could potentially be useful in predicting the effectiveness of immunotherapy, for example, immune checkpoint inhibitors. If we imagine that a patient with a high level of PD-1 negative B cells in the tumor is prescribed inhibitors of immune checkpoints, then the effectiveness will be low.

In this study, we identify a potential simple predictive marker of eribulin response and overall survival in locally advanced or metastatic triple-negative breast cancer patients. But the study was performed on a small cohort of patients and for the clinical application of these results it would be useful to expand the number of observations. Nevertheless, the presented results allow us to think about the fundamental mechanisms of eribulin action and prove the existing immune-related mechanisms.

## Conclusions

PD1-negative-to-PD1-positive B cells ratio in primary tumor more than 3 is an independent predictor of the short-term effectiveness of eribulin and worse overall survival in patients with locally advanced or metastatic triple-negative breast cancer.

## Data Availability Statement

The raw data supporting the conclusions of this article will be made available by the authors, without undue reservation.

## Ethics Statement

The studies involving human participants were reviewed and approved by Tomsk NRMC Institutional Review Board (No. 7 dated April 01, 2019). The patients/participants provided their written informed consent to participate in this study.

## Author Contributions

LT: Methodology, study design, obtaining data, analyzing the data, 34Writing—Original Draft Preparation; NP, AK, VG, EK, EA, AM, DP, NL, ER, SK, and MZ: Provided patients’ data; VP: Writing—Review and Editing. All authors have read and agreed to the published version of the manuscript.

## Funding

The study was supported by Eisai LLC grant. The funder was not involved in the study design, collection, analysis, interpretation of data, the writing of this article or the decision to submit it for publication.

## Conflict of Interest

The authors declare that the research was conducted in the absence of any commercial or financial relationships that could be construed as a potential conflict of interest.

## Publisher’s Note

All claims expressed in this article are solely those of the authors and do not necessarily represent those of their affiliated organizations, or those of the publisher, the editors and the reviewers. Any product that may be evaluated in this article, or claim that may be made by its manufacturer, is not guaranteed or endorsed by the publisher.
